# Methods for Measuring Thermal Conductivity of Two-Dimensional Materials: A Review

**DOI:** 10.3390/nano12040589

**Published:** 2022-02-09

**Authors:** Huanyu Dai, Ridong Wang

**Affiliations:** State Key Laboratory of Precision Measuring Technology and Instruments, Tianjin University, Tianjin 300072, China; huanyu_dai2266@tju.edu.cn

**Keywords:** 2D materials, thermal conductivity, molecular dynamics, Raman spectroscopy

## Abstract

Two-dimensional (2D) materials are widely used in microelectronic devices due to their excellent optical, electrical, and mechanical properties. The performance and reliability of microelectronic devices based 2D materials are affected by heat dissipation performance, which can be evaluated by studying the thermal conductivity of 2D materials. Currently, many theoretical and experimental methods have been developed to characterize the thermal conductivity of 2D materials. In this paper, firstly, typical theoretical methods, such as molecular dynamics, phonon Boltzmann transport equation, and atomic Green’s function method, are introduced and compared. Then, experimental methods, such as suspended micro-bridge, 3ω, time-domain thermal reflectance and Raman methods, are systematically and critically reviewed. In addition, the physical factors affecting the thermal conductivity of 2D materials are discussed. At last, future prospects for both theoretical and experimental thermal conductivity characterization of 2D materials is given. This paper provides an in-depth understanding of the existing thermal conductivity measurement methods of 2D materials, which has guiding significance for the application of 2D materials in micro/nanodevices.

## 1. Introduction

The thermal conductivity of 2D materials is of great significance for both basic research [1,2,3,4,5,6,7,8,9,10] and practical application [11,12,13]. In basic research, Fourier’s law has been successful in studying heat conduction in macroscopic systems. However, when down to micro or nanoscale, due to the existence of size effect [1], thermal rectification [2], and ballistic transport [3], this law is no longer usable. In addition, with the advent of new materials such as newly discovered borophene [4,5], MXene [6,7], and various heterostructures [8,9,10], it is also crucial to determine the thermal conductivity of these new materials. From a practical perspective, 2D materials are widely used in optoelectronic devices [11], biological monitoring [12], and energy storage [13] due to their excellent optical, electrical, and mechanical properties. It is necessary to explore the thermal conductivities of these 2D materials to optimize heat dissipation in optoelectronic devices.

Various theoretical calculation methods such as molecular dynamics simulation [14,15,16,17], phonon Boltzmann transport equation [18,19,20,21,22,23], and atomistic Green’s functions [24,25,26] have been developed to study the underlying physical mechanism of heat transfer in 2D materials. Yet, due to the ignorance of surface defects, the accuracy of these methods is limited. Experimental methods, such as the suspended micro-bridge method [27,28,29,30,31,32], 3ω method [33,34,35,36], time-domain thermoreflectance method [37,38,39,40,41,42,43,44,45,46], and Raman method [47,48,49,50,51,52,53], have been developed to study the thermal conductivity of 2D materials. Bao et al. [54] introduced heat transfer research methods in micro-nano structures from the perspective of theoretical calculation. Experimental-based thermal characterization techniques for low-dimensional materials were also reviewed [55,56]. Considering all these different techniques, however, there is still a lack of comprehensive review on the thermal conductivity measurement methods of 2D materials. In this paper, both theoretical and experimental methods for studying the thermal conductivity of 2D materials are reviewed. In addition, the factors affecting the thermal conductivity of 2D materials are also discussed.

## 2. Theoretical Methods

The theoretical calculation is an effective way to deeply understand the potential mechanism of phonon transport in 2D materials. Currently, the molecular dynamics (MD) simulation, phonon Boltzmann transport equation (PBTE), and atomistic Green’s functions (AGF) were the 3 mainstream theoretical methods.

### 2.1. MD Simulation

In MD simulation, the motion of each particle in the dynamic process was described based on Newton’s second law, and the position, velocity, and force of each atom were calculated at each step. The interatomic forces were derived from the potential function, and the commonly used empirical potential functions were Lennard–Jones (LJ) potential for interlayer van der Waals (vdW) interaction, Stillinger–Weber (SW) [15] potential for atomic interaction, and REBO potential [16] for the covalent bonding of the carbon atoms in diamond and graphite. Two common methods were used to calculate the thermal conductivity of 2D materials: the equilibrium MD (EMD) method based on the Green–Kubo formalism and the nonequilibrium MD (NEMD) method based on Fourier’s law.

In EMD method, the thermal conductivity is expressed as the integration of the heat current autocorrelation function (HCACF) with respect to a given correlation time *t*,

(1) $$\kappa_{\mu v}(t)=\frac{1}{\kappa_{B}T^{2}V}\int_{0}^{t}\langle J_{\mu }(0)J_{v}(t^{\prime })\rangle dt^{\prime }$$

where $\kappa_{B}$ is Boltzmann’s constant, *T* is the absolute temperature of the system, *V* is the volume and $J_{\mu }$ is the μth component of the full heat current vector J. The heat current at a given time depends on the positions and velocities of the particles in the system. The key point of EMD based thermal conductivity calculation is to calculate the time integral with the upper limit of infinity in Equation (1) and ensure its convergence. In addition, the size effect of thermal conductivity is also difficult to be studied in EMD, which can be solved in NEMD. The NEMD technique can be employed to characterize the in-plane thermal conductivity of a sample with finite length L by driving the system out of equilibrium. When steady state is achieved after sufficient time, the heat current (flux) Q and temperature gradient ∇T are obtained to calculate thermal conductivity κ(L) according to Fourier’s law:

(2) $$\kappa (L)=-\frac{Q}{\nabla T}$$

For the MD method, one advantage is that the simulation is based on a real physical model in space, which makes it convenient to study the effects of physical parameters, such as strain, defect, doping, etc. However, the accuracy of MD simulation is highly dependent on the potential empirical function used, which is usually developed by fitting the existing material properties. Moreover, as the MD method is modeled in real space, the calculation range is limited due to the simulation time, which makes the calculation of thermal conductivity not very accurate. In addition, the phonon scattering rate in MD is related to the Maxwell Boltzmann distribution while ignoring the quantum effect below the Debye temperature. Thus, erroneous results were obtained for the calculated thermal conductivity below the Debye temperature [17].

### 2.2. PBTE Method

Recently, PBTE, combined with first principles, was used much more frequently to explore the thermal conductivity of non-metallic materials, such as 2D selinene [18], phosphorene [19], borophane [20], and transition metal dichalcogenide (TMDC) MX_2_ [21]. Under the effect of temperature gradient (∇T), the phonon distribution $f_{\lambda }$ deviates from the Bose–Einstein distribution in equilibrium $f_{\lambda }^{0}$, which can be obtained by solving PBTE:

(3) $${\left.\frac{\partial f_{\lambda }}{\partial t}\right|}_{\mathrm{diff}}+{\left.\frac{\partial f_{\lambda }}{\partial t}\right|}_{\mathrm{scatt}}=0$$

where the diffusion term (${\left.\frac{\partial f_{\lambda }}{\partial t}\right|}_{\mathrm{diff}}$) is caused by the temperature gradient ∇T and given by:

(4) $${\left.\frac{\partial f_{\lambda }}{\partial t}\right|}_{\mathrm{diff}}\;=\;-v_{\lambda }\nabla T\frac{\partial f_{\lambda }^{0}}{\partial T}$$

where $v_{\lambda }$ is the group velocity of phonon mode λ. The scattering term (${\left.\frac{\partial f_{\lambda }}{\partial t}\right|}_{\mathrm{scatt}}$) in Equation (5) is determined by the scattering process in the system. Under the relaxation time approximation (RTA), the scattering term can be written as:

(5) $${\left.\frac{\partial f_{\lambda }}{\partial t}\right|}_{\mathrm{scatt}}\;=\;\frac{f_{\lambda }\;-\;f_{\lambda }^{0}}{\tau_{\lambda }}$$

where $\tau_{\lambda }$ is the relaxation time. Considering anharmonic phonon–phonon interactions $\tau_{\lambda }$ can be obtained by perturbation theory. The heat flow $J^{\alpha }$ in α direction can be written as:

(6) $$J^{\alpha }=\sum_{\lambda }\int \hbar \omega_{\lambda }f_{\lambda }v_{\lambda }^{\alpha }\frac{dk}{2\pi^{3}}$$

where ***k*** denotes the phonon wave vector, $\omega_{\lambda }$ is the frequency of phonon mode. According to Fourier’s law $J^{\alpha }=-\sum_{\beta }\kappa^{\alpha \beta }{\left(\nabla T\right)}^{\beta }$, the lattice thermal conductivity tensor $\kappa^{\alpha \beta }$ under the RTA can be written as:

(7) $$\kappa^{\alpha \beta }=\frac{1}{k_{B}T^{2}NV}\sum_{\lambda }{\left(\hbar \omega_{\lambda }\right)}^{2}f_{\lambda }^{0}(1+f_{\lambda }^{0})v_{\lambda }^{\alpha }v_{\lambda }^{\beta }\tau_{\lambda }$$

where *N* is the total number of phonon wave vectors included in the summation, *V* is the volume of the unit cell, $\nu_{\lambda }^{\alpha }$ and $\nu_{\lambda }^{\beta }$ are the group velocity of phonon mode λ with Cartesian coordinates indexed by α and β, respectively. In the actual simulation, the parameters $\nu_{\lambda }^{\alpha }$ and $\nu_{\lambda }^{\beta }$ were obtained from the interatomic force constants (IFCs), which can be extracted from DFT packages such as VASP. Some open-source software packages such as ShengBTE [22] were available to predict the lattice thermal conductivity of solid materials with the input files of these IFCs.

In PBTE, the calculation accuracy depends on the accuracy of the scattering mechanism in the 2D material. Anharmonicity causes inelastic scattering of phonons. Meanwhile, many factors such as isotopes, holes, and interfaces may disturb the lattice vibration. At present, PBTE lacks the description of some scattering mechanisms, such as holes [23].

### 2.3. AGF Method

**Figure 1:**
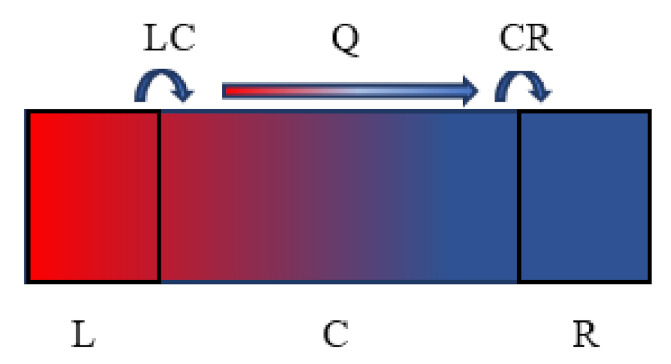
Schematic diagram of heat transport model for low dimensional system. (The L, C and R in the figure represents three parts of the system: central scattering region, left and right lead, and Q indicates the direction of heat flow.).

The AGF method, which is based on a dynamical equation and the quantum mechanical phonon energy distribution, is an effective tool to simulate ballistic transport in nanoscale devices. As shown in Figure 1, the quantum thermal transport system can be divided into 3 parts: central scattering region (abbreviated as C), left and right lead (abbreviated as L, R). Under the harmonic approximation, the phonon waves in the system can be described as:

(8) $$(\omega^{2}I-H)\Phi (\omega )=0$$

where *ω* is the angular frequency of lattice vibration, ***I*** is the identity matrix, ***H*** is the harmonic matrix, and Φ(ω) is the eigenvector of ***H***. The response of the system under small disturbance can be obtained by Green’s function:

(9) $$(\omega^{2}I-H)G=0$$

The atomic interactions in each region are described by constructing a harmonic matrix for AGF calculation [24]. The phonon transmission function Ξ(ω) is calculated by:

(10) $$\Xi (\omega )\;=\;Tr\left[\Gamma_{L}G_{C}^{r}\Gamma_{R}G_{C}^{r}{}^{*}\right]$$

where $G_{C}^{r}$ and $G_{C}^{r}{}^{*}$ are the Green’s function of the central region and its complex conjugate [25], $\Gamma_{L}$ and $\Gamma_{R}$ are phonon escape rates from left contact and right contact, and *Tr* represents the trace of the matrix.

According to the Landauer formula and the phonon transmission function, the thermal conductivity κ of the system can be calculated as:

(11) $$\kappa \;=\;\frac{\hbar }{2\pi }\int_{0}^{\infty }\frac{\partial f(\omega ,T)}{\partial T}\Xi (\omega )\omega d\omega$$

where f(ω,T) is the Bose–Einstein distribution and ℏ is the Planck’s constant. AGF studies the thermal conductivity based on the harmonic approximation condition and does not consider the anharmonic interaction, that is, phonon–phonon scattering. Therefore, AGF, which studies the structure dominated by elastic scattering, is mainly used in nanostructures dominated by harmonic scatterings, such as defects, interfaces, lattice mismatch [26].

## 3. Experimental Methods

The theoretical simulation methods, including MD, PBTE, and AGF, have become effective tools for calculating the thermal conductivity of 2D materials. However, it is challenging to ensure the accuracy when considering the impurities, defects, and rough surface of real samples. That is, it is of great significance to developing experimental methods to improve the measuring accuracy. The measuring accuracy can also be further improved by combining the experimental methods with the theoretical calculation. At present, experimental measurement methods mainly include electro-thermal and optothermal methods.

### 3.1. Electro-Thermal Techniques

Electro-thermal techniques, which include the suspended micro-bridge method, 3ω method, electron beam self-heating, T-bridge, four-probe transport measurements techniques, characterize the thermal conductivities of materials based on the temperature dependence of thermal resistance. The suspended micro-bridge method and 3ω method are two typical techniques and are introduced in detail.

#### 3.1.1. Suspended Micro-Bridge Method

**Figure 2:**
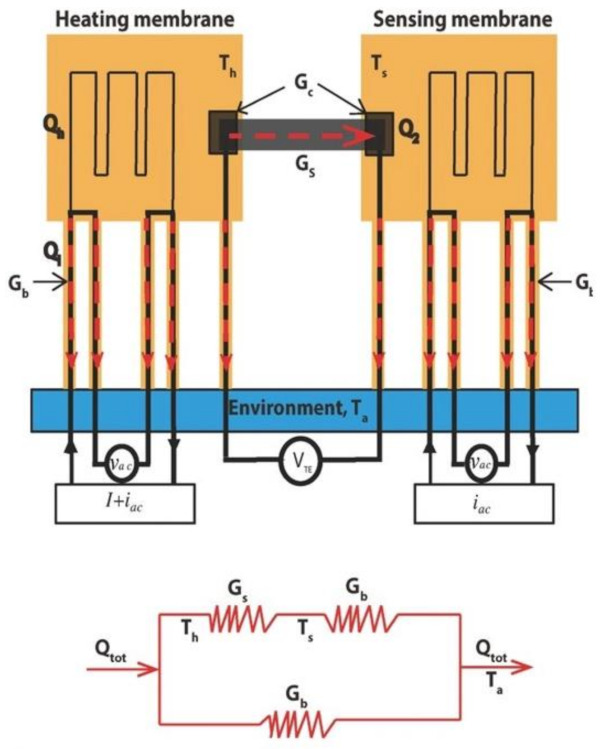
Schematic diagram of microbridge measurement. Reprinted with permission from Ref. [32]. Copyright 2017, John Wiley and Sons.

The suspended micro-bridge method was first used by Majumdar et al. [27,28] in 2001 to measure the thermal conductivity of a single multi-walled nanotube. Since then, the suspended micro-bridge method has been applied to measure the thermal conductivities of graphene [29], hexagonal boron nitride [30], MoS_2_ [31] and other 2D materials. As shown in Figure 2, the suspended device is composed of 2 adjacent silicon nitride (SiNx) membranes suspended by 5 SiNx beams. The platinum resistance thermometer coil designed on each membrane is connected to the substrate through a platinum (Pt) leads on the long SiNx beam. A mixed current of DC (microampere level) and AC (nano ampere level) is introduced to the heating membrane.

The DC current is used to generate Joule heat ($Q_{tot}$) on one side, and the Pt resistance is measured by AC to characterize the temperature change ($\Delta T_{h},\Delta T_{s}$) of heating and sensing membrane caused by $Q_{tot}$. Heat is transferred between the heating membrane and the sensing membrane only through the sample. Since the $Q_{tot}$ is transferred only from the heating membrane to the substrate with an environment temperature *T_a_* and sample, we can express the heat flux distribution on the whole device and sample as follows:

(12) $$Q_{tot}\;=\;Q_{1}\;+\;Q_{2}$$

(13) $$Q_{1}\;=\;G_{b}\;\times \;\Delta T_{h}$$

(14) $$Q_{2}\;=\;G_{s}(\Delta T_{h}\;-\;\Delta T_{s})\;=\;G_{b}\;\times \;\Delta T_{s}$$

where $Q_{tot}$ is the total heat on the heating membrane, $Q_{1}$ and $Q_{2}$ are the heat transferred from the heating membrane to the substrate and the sample, $G_{s}$ and $G_{b}$ are the conductance of the sample and SiNx beams, respectively. The thermal conductivity (κ) of the sample can be written as:

(15) $$\kappa \;=\;G_{s}\frac{L}{S}$$

where *L* and *S* are the length and sectional area of the sample, respectively. In practice, there is thermal resistance ($R_{c}$) at the interface between the sample and the heating/sensing membrane. The measured total thermal resistance is $R\;=\;R_{G}\;+\;2R_{C}$, where $R_{G}$ is the actual thermal resistance of the sample. Some methods have been designed to reduce the effect of $R_{C}$. One is to calculate the temperature rise between the sample and membrane through numerical simulation [30]. In addition, it can be considered to add high thermal conductivity materials to the membrane to reduce $R_{C}$ and improve the uniformity of membrane temperature [29].

#### 3.1.2. 3ω Method

**Figure 3:**
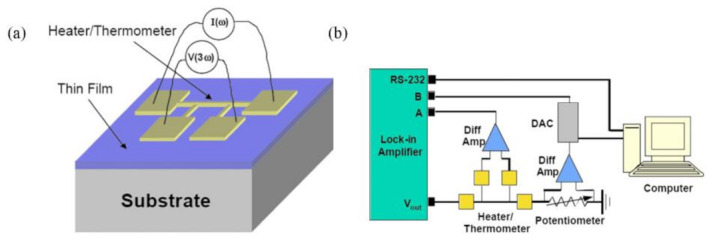
Schematic diagram of (**a**) 3ω method. (**b**) experimental circuit. Reprinted with permission from Ref. [33]. Copyright 2008, AIP Publishing.

The 3ω method is based on the frequency-domain feedback characteristic that the temperature of the heating resistor varies with the frequency of the applied AC electrical current. As shown in Figure 3a, a metal electrode such as Pt with a certain shape and thickness (the yellow part) was prepared on the surface of the thin film sample (the blue part) by photolithography and thermal evaporation, which was used both as a heater and a thermometer. Thin-film samples are usually deposited on the substrate (the bottom gray part) by chemical vapor deposition (CVD) and high-temperature oxidation. When an AC power supply with a frequency of 1ω is connected to the metal electrode, the internal resistance of the metal electrode changes approximately at a frequency of 2ω due to the linear relationship with the temperature change. Finally, the voltage signal with 3ω frequency variation can be extracted by the lock-in amplifier (shown in Figure 3b).

In this technique, 2 structures were prepared: substrate and film-substrate structure. Metal electrodes were deposited on the 2 structures to measure the corresponding temperature changes ($\Delta T_{s}$, $\Delta T_{s\;+\;f}$). Then, temperature change caused by the film can be written as $\Delta T_{f}\;=\;\Delta T_{s\;+\;f}\;-\;\Delta T_{s}$. The thermal conductivity ($\kappa_{f}$) of a thin film is determined using Equation (16)

(16) $$\kappa_{f}\;=\;\frac{Pt}{\Delta T_{f}\;\cdot \;S}$$

where *P* and *t* are heating power and film thickness, respectively.

For the 3ω method, thermal contact resistance measurements between graphene and SiO_2_ based on a differential 3ω technique were made [34]. However, as the fabrication of a metal electrode with high quality and the signal extraction of the phase-locked amplifier were required, it was difficult to measure the thermal conductivity of 2D material with atomic level thickness. Zhang et al. [35] reported the thermal conductivity measurement of 100 nm thickness silicon nitride (SiN) and 64 nm thickness amorphous boron nitride (BN) based on the 3ω method. As the thermal conductivity was obtained by the frequency-dependent temperature oscillation, the 3ω method was free of the effect from contact thermal resistance between sample and substrate. Then, due to the small surface area of metal electrode, the effect of heat radiation was also limited [36]. The 3ω method also has some drawbacks that further limit its application for supported samples. The thermal conductivity of the substrate should be much higher than that of the film deposited on it to ensure a high sensitivity. A lower surface roughness of the sample is needed to prevent damage to the thin metal wires.

### 3.2. Opto-Thermal Techniques

Compared with electrothermal method, opto-thermal techniques, which can realize non-contact measurement with simple sample preparation, have been widely used in thermal conductivity characterization of 2D materials. Two representative methods, time-domain thermoreflectance (TDTR) method, and Raman-based methods, are introduced in detail in this section.

#### 3.2.1. Time-Domain Thermoreflectance (TDTR) Method

**Figure 4:**
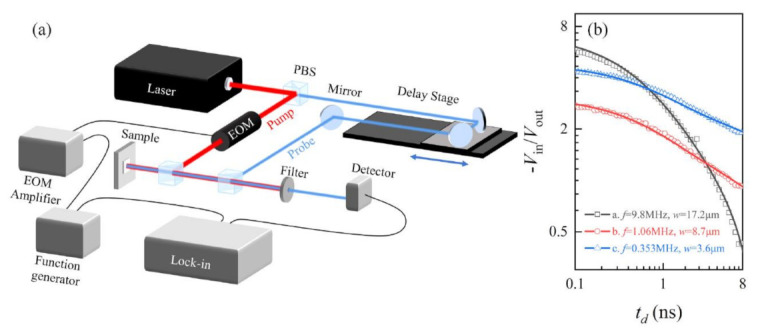
(**a**) Schematic of a typical TDTR setup. (**b**) The ratio between in-phase and out-of-phase signals, −*V*_in_/*V*_out_ as a function of delay time is compared with the thermal modeling to extract the thermal conductivity. Reprinted with permission from Ref. [39]. Copyright 2021, AIP Publishing.

The TDTR method is based on the change of surface reflectance caused by temperature change. Figure 4a shows a typical setup. The emitted laser is divided into pump light and probe light through a polarizing beam splitter (PBS). The pump light modulated by the electro-optic modulator is used to heat the sample surface. The sample surface is usually covered with a metal film in order to ensure that the pump laser is absorbed at the surface. The detection beam is delayed relative to the pump light by the mechanical delay stage and received by the photodiode detector. The converted electrical signal is extracted by the lock-in amplifier with two outputs: in-phase ($V_{\mathrm{in}}$) signal and out-of-phase ($V_{\mathrm{out}}$) signal, which represent the phase of the reflected beam related to the temperature response and can be written as $R\;=\;-V_{\mathrm{in}}/V_{\mathrm{out}}$. By continuously changing the delay time, the curve of R versus time shown in Figure 4b can be obtained. Combined with the heat transfer model established by Cahil et al. [37] in 2004, the thermal conductivity can be extracted. Schmidt et al. [38] further applied the model in anisotropic thermal conduction of highly ordered pyrolytic graphite (HOPG).

In TDTR, due to the deposition of metal films on the sample surface, the intrinsic thermal conductivity of the sample cannot be measured accurately. The main limitation for TDTR is the requirement for a highly smooth surface to minimize diffuse reflection and the complex experimental device. In the later development, many improvements were made based on TDTR, such as FDTR [40] TDTR based on time-resolved magneto-optical Kerr effect (TR-MOKE) [41]. In FDTR, the relationship between thermal reflection signal and modulation frequency rather than the delay time was established, in which continuous laser can be used thus it is simpler and cheaper. TDTR can also be combined with TR-MOKE to probe the sample’s surface temperature, which depends on the temperature dependence of the polarization rather than the intensity of the reflected beam. This temperature measurement method allows us to use thinner ferromagnetic metal film as a transducer, reduces lateral heat flow in the metal film, and improves the accuracy of measurement results. Currently, for thermal conductivity measurement, the TDTR method is mostly used in thin films, such as transition metal dichalcogenides MX_2_ (M = Mo, W and X = S, Se) [42], h-BN [43], BP [44], which requires a relatively large thickness (>100 nm). For 2D materials, this technique can realize the characterization of the interfacial heat transfer [45]. Additionally, FDTR, which is an improved TDTR method, is used in measuring the thermal conductivity of 2D materials. Rahman et al. [46] implemented frequency domain magnetooptical Kerr effect (FD-MOKE) to measure the thermal conductivity of various 2D materials, such as graphene, monolayer MoS_2_, and four-layer h-BN.

#### 3.2.2. Optothermal Raman Methods

Compared with the complex measurement device of TDTR, Raman-based methods are simpler and have been widely used in the thermal conductivity measurement of 2D materials. By constructing different heat transfer states in the time and space domain, various Raman-based measurement methods were developed. Among them, the optothermal Raman method based on steady-state heating is the most commonly used.

**Figure 5:**
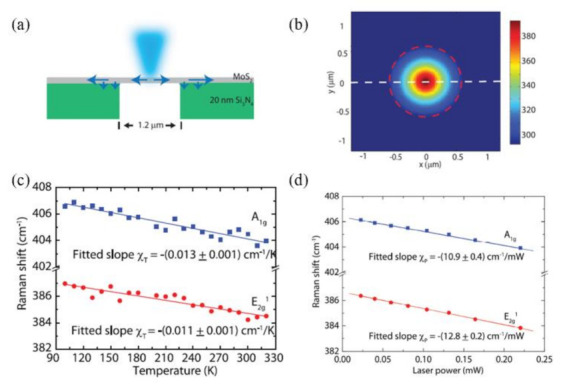
Illustration of optothermal Raman methods. (**a**) Schematic of the thermal conductivity measurement showing suspended MoS_2_ flakes and excitation laser light. (**b**) Simulation of laser heating temperature rise. (**c**) Raman peak frequency shift as a function of temperature. (**d**) Experimental data for Raman shift as a function of laser power, which determines the local temperature rise in response to the dissipated power. Reprinted with permission from Ref. [47]. Copyright 2014, American Chemical Society.

In this method, the sample can be heated optically or electrically. Taking optical heating as an example, Figure 5a shows the MoS_2_ sample is suspended on a Si_2_N_4_ substrate and heated by a focused laser light. The heat can only diffuse around the sample and eventually to the substrate. As shown in Figure 5c, the Raman shift (Δω) of MoS_2_ is linearly related to the local temperature change (ΔT) of the sample upon laser heating, and can be written as: $\Delta \omega \;=\;\chi_{T}\Delta T$, where $\chi_{T}$ is the first-order temperature coefficient. Varying laser power will also produce different thermal effects, which means that there is a similar linear relationship between Raman shift and laser power (Figure 5d). For the sample suspended on a hole with radius *R*, the temperature at *r* from the center of the hole can be calculated from the heat conduction equation as follows:

(17) $$\kappa_{\mathrm{sus}}\frac{1}{r}\frac{d}{dr}\left[r\frac{dT(r)}{dr}\right]\;+\;q(r)\;=\;0,\;\mathrm{for}\;r\;\leq \;R$$

(18) $$\kappa_{\sup}\frac{1}{r}\frac{d}{dr}\left[r\frac{dT(r)}{dr}\right]\;+\;\frac{g}{t}\left[T(r)\;-\;T(a)\right]\;=\;0,\;\mathrm{for}\;r\;\geq \;R$$

where $\kappa_{\mathrm{sus}}$, $\kappa_{\sup}$, q(r),g, t and T(a) are the thermal conductivity of suspended and supported structure, volume optical heating, interface thermal conductivity, thickness of sample and environmental temperature, respectively. Figure 5b shows the calculated temperature distribution of the sample. The weighted average temperature rise in the laser spot can be written as:

(19) $$T_{calculated}\;\approx \;\frac{\int_{0}^{R}T(r)q(r)rdr}{\int_{0}^{R}q(r)rdr}$$

By matching the calculated temperature rise ($T_{calculated}\;-\;T_{a}$) with the temperature rise measured by Raman spectroscopy ($\Delta T_{measured}$), the thermal conductivity κ can be extracted.

One drawback of optothermal Raman method is the measurement of absolute laser absorption power. Under laser heating, part of the energy is absorbed by the sample, while the rest of the energy is reflected by the sample or transmitted to the substrate. Currently, it is very difficult to determine the laser absorption coefficient accurately. In addition, a temperature calibration process, which is time-consuming and can introduce large errors, is also needed.

#### 3.2.3. Time-Resolved Raman Methods

**Figure 6:**
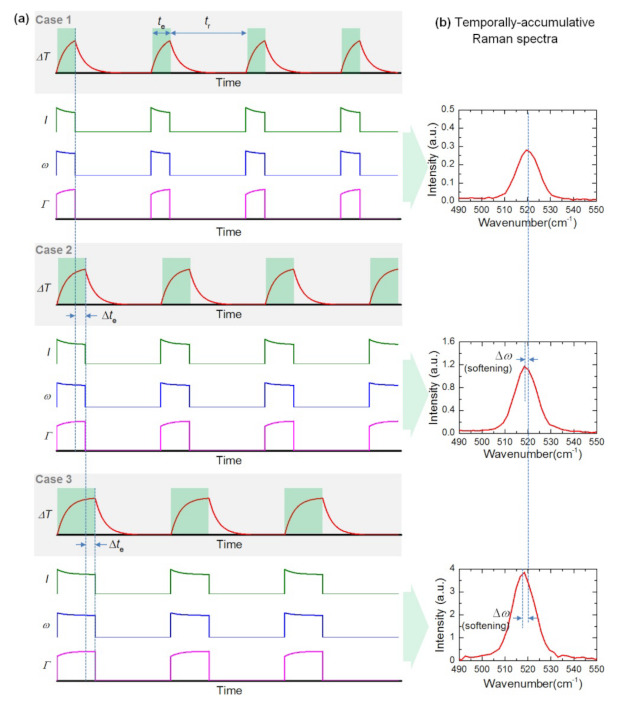
Concept of TD−Raman. (**a**) The change of temperature evolution(∆T), and instant changes of Raman peak intensity (I), peak shift (ω) and linewidth (Γ). (**b**) The corresponding temporally accumulative Raman spectra of one laser pulse cycle in Case 1, 2, and 3. Reprinted with permission from Ref. [48] © The Optical Society. Copyright 2015, The Optical Society.

Time-domain differential Raman (TD-Raman) [48], which uses a square wave modulated laser with variable duty cycle, can be applied to measure the thermal conductivity of 2D materials. As shown in Figure 6a, the modulated laser is used for sample heating and Raman excitation, which consists of a variable excitation period $t_{e}$ and a fixed thermal relaxation period $t_{r}$. Here, the thermal relaxation time $t_{r}$ needs to be long enough for the sample to cool completely before the next pulse period. Figure 6b shows the corresponding temporally accumulative Raman spectra of one laser pulse cycle in 3 cases. It can be seen that longer excitation time $t_{e}$ leads to higher temperature rise, and the corresponding Raman spectra also change. From cases 1 to 3, the intensity of the Raman peak increases gradually, and the softening phenomenon of Raman peak position is also observed. By analyzing the changes of Raman signals mentioned above, the average temperature rise ($\Delta \bar{T}$) of the sample in the heating zone can be determined by Raman spectroscopy. Moreover, the accumulative Raman emission for one excitation cycle (0~$t_{e}$) is written as:

(20) $$E_{\omega }(\omega ,t_{e})=I_{0}\int_{0}^{t_{e}}\left(1-A\Delta {\bar{T}}^{*}\right)\;\exp\left[-\frac{4\ln2\cdot {\left(\omega -\omega_{0}+B\Delta {\bar{T}}^{*}\right)}^{2}}{{\left(\Gamma_{0}+C\Delta {\bar{T}}^{*}\right)}^{2}}\right]dt$$

where $I_{0}$, $\omega_{0}$, $\Gamma_{0}$ are the corresponding Raman properties at the beginning of laser heating, A, B, C are the changing rate of Raman intensity, Raman shift, and linewidth against the normalized temperature $\Delta {\bar{T}}^{*}$. Combining with the transient heat transfer model, the thermal conductivity of the sample can be determined by fitting the variation of Raman peak with time.

**Figure 7:**
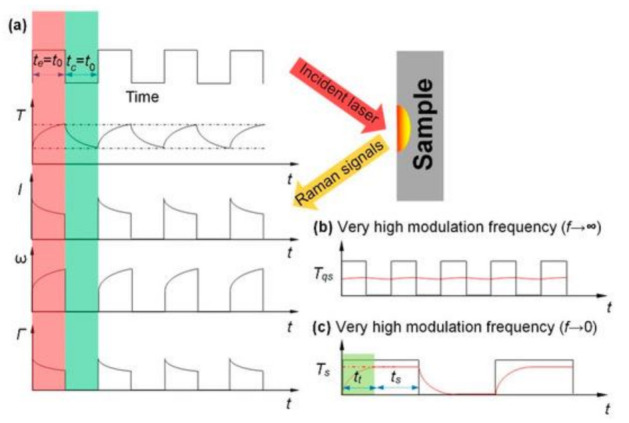
Concept of FR-Raman. (**a**) Time profiles of laser pulse, temperature evolution (*T*), Raman peak intensity (*I*), peak shift (*ω*) and linewidth (*Γ*). (**b**) Temperature variation (*T_QS_*) at quasi-steady state. (**c**) Temperature variation (*T_S_*) at very low frequency. Reprinted with permission from [49] © The Optical Society. Copyright 2016, The Optical Society.

In TD-Raman, thermal conductivity of 2D materials is measured through the Raman characterization of transient heat transfer. However, in practice, when the heating time is too short, a long time of Raman signal acquisition is needed, which makes it hard for fast thermal transport characterization produces more environmental interference and affects the measurement accuracy. To solve this problem, Wang’s lab further developed frequency-resolved (FR) Raman technique Frequency-resolved Raman for transient thermal probing and thermal diffusivity measurement [49]. As shown in Figure 7, an amplitude-modulated square-wave with different frequencies is employed to heat the sample and excite Raman signals. When the sample is irradiated by the high-frequency laser pulse, the temperature of the sample is almost constant in the whole process, which is defined, as “quasi-steady state,” and the temperature rise is regarded as $\Delta T_{qs}$. On the contrary, when low-frequency laser pulse irradiates the sample because the sample has enough time to rise to a stable state in the excitation time, the temperature of the sample is approximately regarded as a constant in the excitation time, which is defined as “steady state,” and the temperature rise is regarded as $\Delta T_{s}$. Here we have $\Delta T_{qs}\;=\;\Delta T_{s}/2$, which shows that the temperature decreases with increasing frequency. The thermal conductivity can be extracted based on the transient heat transfer model and the collected Raman signal. TD-Raman has been applied to the measurement of the anisotropic thermal conductivity of black phosphorus [50]. Compared with TD-Raman, the Raman signal collection of FR-Raman is more efficient, but the sensitivity is lower because the time between pulses is not enough to completely cool the sample.

#### 3.2.4. Energy Transport State Resolved Raman (ET-Raman)

**Figure 8:**
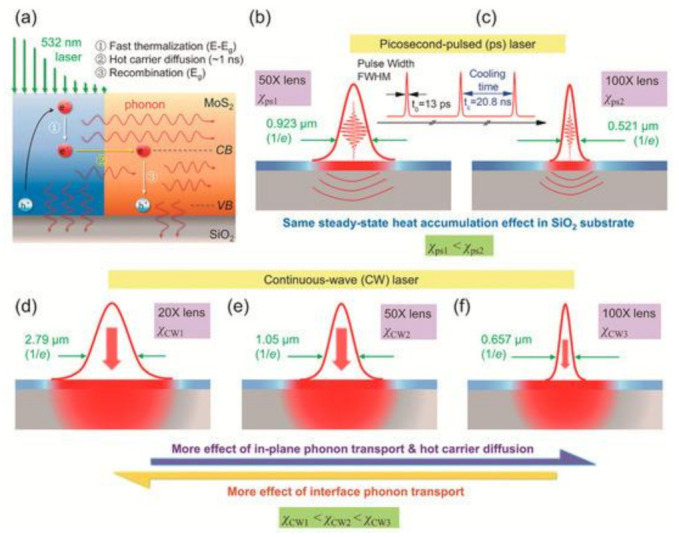
The Schematic diagram for mechanism of five-state energy transport state-resolved Raman (ET-Raman) technique. (**a**) The generation, diffusion, and recombination of the hot carrier in MoS_2_ upon laser irradiating. (**b**,**c**) Transient state heating using picosecond laser heating under 50× and 100× objective lenses. (**d**–**f**) Steady state heating using CW laser with 20×, 50×, and 100× objective lenses. Reproduced from Ref. [51] with permission from the Royal Society of Chemistry. Copyright 2018, Royal Society of Chemistry.

Besides the time-domain modulation, the energy transport states can also be modulated in the spatial domain. Based on this, a technique named ET-Raman was developed to measure the in-plane thermal conductivities of supported or suspended 2D materials. For supported 2D samples, both a CW laser and a picosecond laser are used. As shown in Figure 8, 5 energy transport states were constructed both in time and spatial domains [51]. Three physical processes occur with laser heating. The first is hot carrier generation, diffusion in space, and electron–hole recombination. This process introduces heat transfer and energy redistribution, which is determined by the hot carrier diffusivity (*D*). The subsequent process is the heat conduction by phonons, which receives energy from the hot carriers or electron–hole recombination, which mainly happens in the in-plane and depends on the thermal conductivity (*κ*). The third is the heat conduction from sample to substrate, and this process is dominated by the local thermal resistance (*R*).

**Figure 9:**
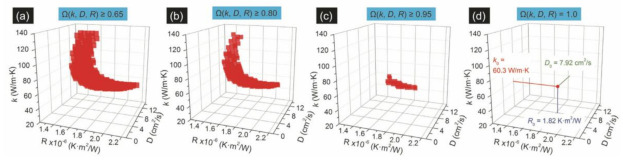
The evolution of distribution of Ω(κ,D,R). (**a**) Ω(κ,D,R)≤0.65; (**b**) Ω(κ,D,R)≥0.80; (**c**) Ω(κ,D,R)≥0.95; (**d**) Ω(κ,D,R) = 1.0. Reproduced from Ref. [51] with permission from the Royal Society of Chemistry.

By using different laser power (*P*), a parameter named Raman shift power coefficient (RSC) can be obtained and expressed as: χ = ∂ω/∂P, where ω is Raman peak shift. Moreover, χ is determined by κ, *D*, *R*, laser absorption coefficient and temperature coefficient of Raman shift. According to the 5 heating states in Figure 8, 3 normalized RSC were obtained: $\Theta_{n}\;=\;\chi_{cw,n}/(\chi_{ps1}\;-\;\chi_{ps2}),\;n\;=\;1,2,3$. The error caused by laser absorption, Raman temperature coefficient were eliminated. Meanwhile, the heat accumulation effect was removed by the difference of the heating between the 2 objectives (50×, 100×) under picosecond pulse laser. Then, a 3D numerical model was employed to determine κ, *D* and *R*. Figure 9 shows the evolution of the distribution of Ω(κ, *D*, *R*). Yuan et al. measured that the in-plane thermal conductivity spans from 31.0 to 76.2 W/(m·K) of 2D few layers MoS_2_ samples (thickness ranging from 2.4 nm to 37.8 nm) supported on a glass substrate by 5 state picosecond ET-Raman method.

**Figure 10:**
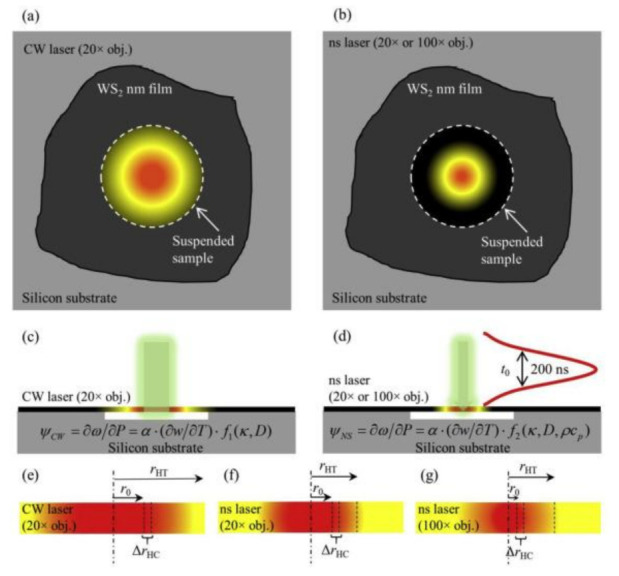
(**a**,**b**) Schematic diagram of suspended WS_2_ illuminated by continuous and nanosecond lasers. (**c**,**d**) Energy transport states are constructed by continuous and nanosecond lasers in the temporal and spatial domain. (**e**–**g**) Thermal diffusion length, laser radius, and carrier diffusion length under three states. Reprinted with permission from Ref. [52]. Copyright 2019, Elsevier.

Due to the short pulse interval, the picosecond laser, which would generate heat accumulation in the suspended structure, is replaced by a nanosecond laser. As shown in Figure 10, Zobeiri et al. measured the κ and D of suspended WS_2_ by constructing 3 heating states with a continuous laser and a nanosecond pulse laser [52]. The influence of κ and D can be distinguished by changing the size of the heating area with a different objective lens. Similarly, 3 RSCs were defined: $\psi_{CW}$, $\psi_{ns20}$ and $\psi_{ns100}$. Two normalized RSCs were further defined: $\Theta_{20}\;=\;\psi_{ns20}/\psi_{CW}$ and $\Theta_{100}\;=\;\psi_{ns100}/\psi_{CW}$. Theoretical values Θ under different κ and *D* values were obtained by temperature rise simulation under 3 states, and the matching κ and *D* were obtained by comparing with the experimental values. The thermal conductivity of suspended WS_2_ was observed to increase from 15.1 to 38.8 W/(m·K) as the sample thickness increased from 13 nm to 107 nm with nanosecond ET-Raman technique.

**Figure 11:**
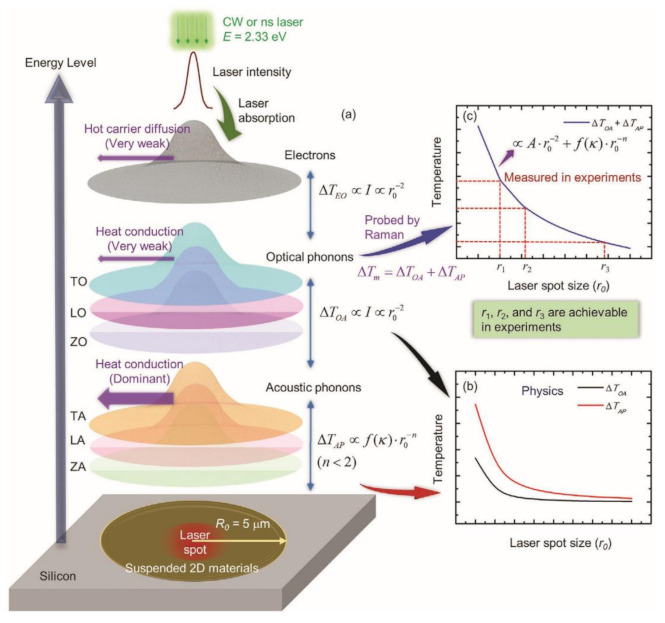
(**a**) Energy transport process among different energy carriers in suspended 2D materials under laser irradiation. (**b**) The temperature difference between OP and AP against laser spot size. (**c**) Acquisition of thermal conductivity of 2D materials and coupling coefficient between OP and AP. Reprinted with permission from Ref. [53]. Copyright 2020, John Wiley and Sons.

Since the Raman signal comes from optical phonons (OPs), but the heat transfer in the sample is related to acoustic phonons (APs). Considering the measurement error caused by ignoring the temperature difference between the 2 phonons, Wang et al. developed 6 heating states nanosecond ET-Raman technique by changing the objective lens: 3 steady states and 3 transient states, and realized the measurement of intrinsic *κ* of MoS_2_ and MoSe_2_ nanofilms and phonon coupling factors [53]. As shown in Figure 11a, the Raman spectrum reflects the temperature rise ($\Delta T_{m}$) of OPs, which is the sum of the temperature difference ($\Delta T_{OA}$) between OPs and APs and the temperature rise ($\Delta T_{AP}$) of APs. Figure 11b shows that the $\Delta T_{\mathrm{OA}}$ decreases to zero faster than $\Delta T_{\mathrm{AP}}$, which means that phonon coupling between OPs and APs is negligible when the laser spot is very large. Figure 11c shows the $\Delta T_{m}$ with different laser spot radius, which can be written as:

(21) $$\Delta T_{m}\;=\;\Delta T_{\mathrm{OA}}\;+\;\Delta T_{\mathrm{AP}}\;\propto \;Ar_{0}^{-2}\;+\;f(\kappa )\;\cdot \;r_{0}^{-n}\;(n\;<\;2)$$

where $r_{0}$ is the laser spot radius, and f(κ) is a function of thermal conductivity κ. Based on this more accurate temperature rise fitting process, the intrinsic thermal conductivity of the sample is approximately extracted.

In order to compare the different experimental methods for measuring the thermal conductivity of 2D materials much more conveniently, these methods are summarized in Table 1. The thermal conductivity values of 2D materials with a similar thickness measured by different experimental methods were quite different, which may be attributed to the differences in sample quality and different measurement methods.

## 4. Analysis of Factors Affecting Thermal Conductivity of 2D Materials

The thermal conductivity of 2D materials is affected by many factors, such as length, thickness, temperature, substrate, strain, and so on. These factors will affect the process of phonon transmission and scattering and further affect the thermal conductivity.

### 4.1. Size Effect

Unlike bulk materials, the thermal conductivity of nm-thick 2D materials usually exhibits an abnormal size dependence. In Zhang’s work [59], the in-plane thermal conductivity of h-BCN monolayer calculated by NEMD increases with sample length increasing from 10 nm to 250 nm.

The factor for the size-dependent thermal conductivity originates in phonon scatterings at the sample boundaries. When the phonon mean free path (*λ*) is larger than the length (l) of the system, heat transfer is ballistic. Certain phonon modes can transmit from the heat-source to the heat-sink without scattering. When l>λ, phonon scattering is suppressed. Therefore the calculated κ results change with length *l* on small scales. In order to extract the thermal conductivity $\kappa_{\infty }$ in an infinitely long system, Schelling et al. [60] proposed an extrapolation formula:

(22) $$\frac{1}{\kappa (l)}\;=\;\frac{1}{\kappa_{\infty }}(1\;+\;\frac{\lambda }{l})$$

### 4.2. Thickness Effect

The thermal conductivity of 2D materials is also thickness-dependent. In the work of Smith et al. [61], the thermal conductivity of BP was observed to increase with the thickness increasing from 10 to 1000 nm. However, when the thickness is reduced to less than 10 nm, the thickness dependence of thermal conductivity may show the opposite trend. Yuan et al. [51] reported that the thermal conductivity of 1 to 10 layers decreases with the increase of layers. All these are related to different phonon scattering modes. In monolayer materials, the thermal conductivity is mostly affected by the boundary scattering. Moreover, Umklapp scattering is quenched. Nevertheless, Umklapp scattering has a more significant effect in thicker materials with long phonon mean free path, and the boundary scattering effect is weak which leads to low thermal conductivity.

### 4.3. Temperature Effect

Temperature, which can directly affect the thermal performances and cause adverse effects on the structural stability of 2D materials, is also an important factor affecting κ. Hong et al. [62] studied κ of phosphorene/graphene under different temperatures using NEMD. The result showed that the κ of phosphorene and graphene decreased with the increase of temperature, which was as expected for phonon-dominated crystalline materials. As the system temperature increases, more high-frequency phonons are activated, which accelerates heat conduction. Meanwhile, high temperature also promotes Umklapp scattering, which suppresses phonon transmission. The strong scattering effect plays a leading role in the process of heat transfer and eventually leads to the decrease of thermal conductivity. The results show that the maximum reduction of thermal conductivity κ of phosphorene and graphene from 100 K to 400 K is, respectively, 64%, 58%, 11%, and 13%. The calculated thermal conductivity is inversely proportional to the temperature, indicating that the Umklapp scattering is dominant in the temperature range.

### 4.4. Other Influence Factors

As the main heat carrier in 2D materials, the propagation of phonons can be adjusted by many other factors, such as lattice deformation caused by strain, substrate coupling, isotope-engineering, which leads to the change of thermal conductivity. Zhang et al. reported that a small strain has a positive effect on the heat conduction of monolayer h-BCN [59]. With further stretching, the thermal conductivity of h-BCN monolayer begins to decrease. Chen et al. reported that the thermal conductivity of SLG supported on amorphous SiO_2_ substrate decreased by 40% compared with suspended SLG structure. Through spectral energy density (SED) analysis, it was found that substrate coupling inhibits the thermal transmission of ZA phonons, resulting in a significant reduction in thermal conductivity [63]. In addition, isotope engineering can also affect the thermal conductivity of 2D materials. Through photothermal Raman measurement, Li et al. reported that the in-plane thermal conductivity of isotopic pure ^100^MoS_2_ monolayer was 50% higher than that synthesized from naturally abundant isotope mixtures. They attribute this to the former having fewer defects, which reduces phonon-defect scattering [64].

## 5. Conclusions and Outlook

In this paper, we systematically introduce the theoretical and experimental methods for the thermal conductivity measurement of 2D materials. The basic principles, advantages, and disadvantages are discussed in detail. Furthermore, some factors (size, temperature, thickness, strain, substrate, and isotope-engineering) that affect the thermal conductivity of 2D materials are also introduced. Based on the thorough analysis, there are many works to conduct to further develop the theoretical and experimental methods.

For the theoretical methods, the accuracy can be further improved by taking more parameters of actual materials into consideration. For example, the growth of 2D materials obtained by chemical vapor deposition (CVD) is controlled by macro physical conditions and parameters, such as partial pressures of each gas in the CVD environment, substrate, defects, furnace configuration, temperature conditions, and gas-phase reactions. One idea is to use growth kinetics and parameter settings to establish the growth model for describing the growth mechanism of the material, which makes the established model consistent with the actual growth model. Netto et al. have reported the continuous growth process of CVD diamond films using time-dependent Monte Carlo algorithm with the chemical reaction mechanism [65]. Recently, due to the high calculation requirements for theoretical methods, machine learning has been employed to accelerate the estimation of material thermal conductivity while ensuring the accuracy of measurement results. Mortazavi et al. [66] employed machine-learning interatomic potentials (MLIPs) trained over short ab initio molecular dynamics (AIMD) trajectories instead of density functional theory (DFT) calculation to evaluate anharmonic interatomic force constants, examining the thermal conductivity conveniently, efficiently, and accurately.

For experimental methods, there is also a lot of work to conduct. For suspended micro bridge devices, the contact thermal resistance is an important factor affecting the accuracy of measurement results, which is quantified in the subsequent development of electron beam self-heating method [67]. Furthermore, the suspended device can combine with TDTR for an ultrafast heat pump and probe. In this way, the influence of contact thermal resistance can be eliminated. Moreover, the non-diffusion heat transfer in 2D materials can be characterized. For the 3ω method, in order to realize its application in measuring the thermal conductivity of 2D materials, the fabrication of a metal electrode with high quality and the signal extraction of a phase-locked amplifier should be considered. Compared with other methods, the Raman method is more widely used in the measurement of 2D material thermal conductivity. However, there is still room for further improvement. First, higher spectral resolution means more accurate temperature measurement. For Raman spectrometer, the higher the grating line density, the higher the corresponding spectral resolution. The grating line density of the commonly used Raman spectrometer ranges from 300 g/mm to 1800 g/mm. If higher density gratings, such as 2400 g/mm and 3600 g/mm, are used, the temperature measurement accuracy will be improved accordingly. Besides, the spatial modulation of laser spot can be considered more. Nowadays, the modulation of laser spot size is realized by using objective lenses with different magnification. In addition, the shape of the laser spot can also be modulated to measure the thermal conductivity of anisotropic 2D materials. Moreover, aside from temporal and spatial modulation, the excitation energy can also be modulated by lasers with different wavelengths. There are few reports on the thermal conductivity of 2D materials using larger wavelength lasers such as 660 nm laser in the visible light band due to its long exposure time and low excitation efficiency. However, at the same time, the long-wavelength laser also has some advantages, such as reducing fluorescence interference and not easily damaging the sample. Therefore, its application in 2D heat transfer measurement is expected.

## Figures and Tables

**Table 1:** Application and comparison of various experimental methods.

Methods	Physical Structure of Materials	Thermal Conductivity	Limitations
Suspended Micro-Bridge	Suspended	Single-layer CVD graphene [29]: 1680 ± 180 Wm^−1^K^−1^	Difficult micro-device preparation, existence of contact thermal resistance
		Bilayer h-BN [30]: $484_{-24}^{+141}$ Wm^−1^K^−1^	
		4L MoS_2_ [31]: 44~50 Wm^−1^K^−1^	
3ω	Supported	100 nm SiN [35]: ~5 Wm^−1^K^−1^ 64 nm BN [35]: ~4 Wm^−1^K^−1^	Not applicable to few layer 2D material, deposition of metal electodes
TDTR	Supported	Single layer graphene ^1^ [46]: 636 ± 140 Wm^−1^K^−1^	Complex experimental device, deposition of a metal film, not applicable to few layer 2D material
Optothermal Raman	Supported and suspended	Single layer graphene [57]: ~4840 to 5300 Wm^−1^K^−1^	The inaccurate measurement results caused by laser absorption coefficient and temperature coefficient calibration
		Few-layer h-BN [58]: 227 to 280 Wm^−1^K^−1^	
		Single layer MoS_2_ [47]: 34.5 ± 4 Wm^−1^K^−1^	
ET-Raman	Supported and suspended	55 nm MoS_2_ [53]: 46.9 ± 3.1 Wm^−1^K^−1^	

^1^ It is measured by the variation of TDTR: FDTR.

## Data Availability

Not applicable.

## References

[1] Kuang Y., Lindsay L., Shi S., Wang X., Huang B. (2016). Thermal conductivity of graphene mediated by strain and size. Int. J. Heat Mass Transf..

[2] Yousefi F., Khoeini F., Rajabpour A. (2020). Thermal conductivity and thermal rectification of nanoporous graphene: A molecular dynamics simulation. Int. J. Heat Mass Transf..

[3] Liu F., Wang Y., Liu X., Wang J., Guo H. (2014). Ballistic transport in monolayer black phosphorus transistors. IEEE Trans. Electron. Devices.

[4] Mannix A.J., Zhang Z., Guisinger N.P., Yakobson B.I., Hersam M.C. (2018). Borophene as a prototype for synthetic 2D materials development. Nat. Nanotechnol..

[5] Peng B., Zhang H., Shao H., Xu Y., Zhang R., Zhu H. (2016). The electronic, optical, and thermodynamic properties of borophene from first-principles calculations. J. Mater. Chem. C.

[6] Chen L., Shi X., Yu N., Zhang X., Du X., Lin J. (2018). Measurement and analysis of thermal conductivity of Ti3C2Tx MXene films. Materials.

[7] Zha X.-H., Zhou J., Zhou Y., Huang Q., He J., Francisco J.S., Luo K., Du S. (2016). Promising electron mobility and high thermal conductivity in Sc 2 CT 2 (T = F, OH) MXenes. Nanoscale.

[8] Mortazavi B., Podryabinkin E.V., Roche S., Rabczuk T., Zhuang X., Shapeev A.V. (2020). Machine-learning interatomic potentials enable first-principles multiscale modeling of lattice thermal conductivity in graphene/borophene heterostructures. Mater. Horiz..

[9] Rahman M.H., Islam M.S., Islam M.S., Chowdhury E.H., Bose P., Jayan R., Islam M.M. (2021). Phonon thermal conductivity of the stanene/hBN van der Waals heterostructure. Phys. Chem. Chem. Phys..

[10] Mayelifartash A., Abdol M.A., Sadeghzadeh S. (2021). Thermal conductivity and interfacial thermal resistance behavior for the polyaniline–boron carbide heterostructure. Phys. Chem. Chem. Phys..

[11] Wang X., Cui Y., Li T., Lei M., Li J., Wei Z. (2019). Recent advances in the functional 2D photonic and optoelectronic devices. Adv. Opt. Mater..

[12] Munteanu R.-E., Moreno P.S., Bramini M., Gáspár S. (2020). 2D materials in electrochemical sensors for in vitro or in vivo use. Anal. Bioanal. Chem..

[13] Dong Y., Wu Z.-S., Ren W., Cheng H.-M., Bao X. (2017). Graphene: A promising 2D material for electrochemical energy storage. Sci. Bull..

[14] Krishnamoorthy A., Rajak P., Norouzzadeh P., Singh D.J., Kalia R.K., Nakano A., Vashishta P. (2019). Thermal conductivity of MoS_2_ monolayers from molecular dynamics simulations. AIP Adv..

[15] Kandemir A., Yapicioglu H., Kinaci A., Çağın T., Sevik C. (2016). Thermal transport properties of MoS_2_ and MoSe_2_ monolayers. Nanotechnology.

[16] Brenner D.W., Shenderova O.A., Harrison J.A., Stuart S.J., Ni B., Sinnott S.B. (2002). A second-generation reactive empirical bond order (REBO) potential energy expression for hydrocarbons. J. Phys. Condens. Matt..

[17] Khan A.I., Navid I.A., Noshin M., Uddin H.M.A., Hossain F.F., Subrina S. (2015). Equilibrium molecular dynamics (MD) simulation study of thermal conductivity of graphene nanoribbon: A comparative study on MD potentials. Electronics.

[18] Liu G., Gao Z., Li G.-L., Wang H. (2020). Abnormally low thermal conductivity of 2D selenene: An ab initio study. J. Appl. Phys..

[19] Hong Y., Zhang J., Zeng X.C. (2018). Thermal transport in phosphorene and phosphorene-based materials: A review on numerical studies. Chinese Phys. B.

[20] Liu G., Wang H., Gao Y., Zhou J., Wang H. (2017). Anisotropic intrinsic lattice thermal conductivity of borophane from first-principles calculations. Phys. Chem. Chem. Phys..

[21] Zulfiqar M., Zhao Y., Li G., Li Z., Ni J. (2019). Intrinsic Thermal conductivities of monolayer transition metal dichalcogenides MX 2 (M = Mo, W.; X = S, Se, Te). Sci. Rep..

[22] Li W., Carrete J., Katcho N.A., Mingo N. (2014). ShengBTE: A solver of the Boltzmann transport equation for phonons. Comput. Phys. Commun..

[23] Bouzerar G., Thébaud S., Pecorario S., Adessi C. (2020). Drastic effects of vacancies on phonon lifetime and thermal conductivity in graphene. J. Phys. Condens. Matt..

[24] Zhang W., Fisher T.S., Mingo N. (2007). The atomistic green’s function method: An efficient simulation approach for nanoscale phonon transport. Num. Heat Transf. Part B Fundam..

[25] Zhang W., Fisher T., Mingo N. (2007). Simulation of interfacial phonon transport in Si–Ge heterostructures using an atomistic Green’s function method. J. Heat Transf..

[26] Li X., Yang R. (2012). Effect of lattice mismatch on phonon transmission and interface thermal conductance across dissimilar material interfaces. Phys. Rev. B.

[27] Kim P., Shi L., Majumdar A., McEuen P.L. (2001). Thermal transport measurements of individual multiwalled nanotubes. Phys. Rev. Lett..

[28] Shi L., Li D., Yu C., Jang W., Kim D., Yao Z., Kim P., Majumdar A. (2003). Measuring thermal and thermoelectric properties of one-dimensional nanostructures using a microfabricated device. J. Heat Transf..

[29] Jo I., Pettes M.T., Lindsay L., Ou E., Weathers A., Moore A.L., Yao Z., Shi L. (2015). Reexamination of basal plane thermal conductivity of suspended graphene samples measured by electro-thermal micro-bridge methods. AIP Adv..

[30] Wang C., Guo J., Dong L., Aiyiti A., Xu X., Li B. (2016). Superior thermal conductivity in suspended bilayer hexagonal boron nitride. Sci. Rep..

[31] Jo I., Pettes M.T., Ou E., Wu W., Shi L. (2014). Basal-plane thermal conductivity of few-layer molybdenum disulfide. Appl. Phys. Lett..

[32] Wang Y., Xu N., Li D., Zhu J. (2017). Thermal properties of two dimensional layered materials. Adv. Funct. Mater..

[33] Liu C.-K., Yu C.-K., Chien H.-C., Kuo S.-L., Hsu C.-Y., Dai M.-J., Luo G.-L., Huang S.-C., Huang M.-J. (2008). Thermal conductivity of Si/SiGe superlattice films. J. Appl. Phys..

[34] Chen Z., Jang W., Bao W., Lau C.N., Dames C. (2009). Thermal contact resistance between graphene and silicon dioxide. Appl. Phys. Lett..

[35] Zhang D., Behbahanian A., Roberts N.A. (2020). Thermal conductivity measurement of supported thin film materials using the 3$\omega $ method. arXiv.

[36] Cahill D.G. (1990). Thermal conductivity measurement from 30 to 750 K: The 3ω method. Rev. Sci. Instrum..

[37] Cahill D.G. (2004). Analysis of heat flow in layered structures for time-domain thermoreflectance. Rev. Sci. Instrum..

[38] Schmidt A.J., Chen X., Chen G. (2008). Pulse accumulation, radial heat conduction, and anisotropic thermal conductivity in pump-probe transient thermoreflectance. Rev. Sci. Instrum..

[39] Pang Y., Jiang P., Yang R. (2021). Machine learning-based data processing technique for time-domain thermoreflectance (TDTR) measurements. J. Applied Phys..

[40] Schmidt A.J., Cheaito R., Chiesa M. (2009). A frequency-domain thermoreflectance method for the characterization of thermal properties. Rev. Sci. Instrum..

[41] Liu J., Choi G.-M., Cahill D.G. (2014). Measurement of the anisotropic thermal conductivity of molybdenum disulfide by the time-resolved magneto-optic Kerr effect. J. Appl. Phys..

[42] Jiang P., Qian X., Gu X., Yang R. (2017). Probing anisotropic thermal conductivity of transition metal dichalcogenides MX2 (M = Mo, W and X = S, Se) using time-Domain thermoreflectance. Adv. Mater..

[43] Wang Y., Xu L., Yang Z., Xie H., Jiang P., Dai J., Luo W., Yao Y., Hitz E., Yang R. (2018). High temperature thermal management with boron nitride nanosheets. Nanoscale.

[44] Jang H., Wood J.D., Ryder C.R., Hersam M.C., Cahill D.G. (2015). Anisotropic thermal conductivity of exfoliated black phosphorus. Adv. Mater..

[45] Lu B., Zhang L., Balogun O. (2019). Cross-plane thermal transport measurements across CVD grown few layer graphene films on a silicon substrate. AIP Adv..

[46] Rahman M., Shahzadeh M., Pisana S. (2019). Simultaneous measurement of anisotropic thermal conductivity and thermal boundary conductance of 2-dimensional materials. J. Appl. Phys..

[47] Yan R., Simpson J.R., Bertolazzi S., Brivio J., Watson M., Wu X., Kis A., Luo T., Hight Walker A.R., Xing H.G. (2014). Thermal Conductivity of Monolayer Molybdenum Disulfide Obtained from Temperature-Dependent Raman Spectroscopy. ACS Nano.

[48] Xu S., Wang T., Hurley D., Yue Y., Wang X. (2015). Development of time-domain differential Raman for transient thermal probing of materials. Opt. Express.

[49] Wang T., Xu S., Hurley D.H., Yue Y., Wang X. (2016). Frequency-resolved Raman for transient thermal probing and thermal diffusivity measurement. Opt. Lett..

[50] Wang T., Han M., Wang R., Yuan P., Xu S., Wang X. (2018). Characterization of anisotropic thermal conductivity of suspended nm-thick black phosphorus with frequency-resolved Raman spectroscopy. J. Appl. Phys..

[51] Yuan P., Wang R., Wang T., Wang X., Xie Y. (2018). Nonmonotonic thickness-dependence of in-plane thermal conductivity of few-layered MoS 2: 2.4 to 37.8 nm. Phys. Chem. Chem. Phys..

[52] Zobeiri H., Wang R., Zhang Q., Zhu G., Wang X. (2019). Hot carrier transfer and phonon transport in suspended nm WS2 films. Acta Mater..

[53] Wang R., Zobeiri H., Xie Y., Wang X., Zhang X., Yue Y. (2020). Distinguishing optical and acoustic phonon temperatures and their energy coupling factor under photon excitation in nm 2D materials. Adv. Sci..

[54] Bao H., Chen J., Gu X., Cao B. (2018). A review of simulation methods in micro/nanoscale heat conduction. ES Energy Environ..

[55] Liu J., Li P., Zheng H. (2021). Review on techniques for thermal characterization of graphene and related 2D materials. Nanomaterials.

[56] Gu X., Yang R. (2016). Phonon transport and thermal conductivity in two-dimensional materials. Ann. Rev. of Heat Transf..

[57] Balandin A.A., Ghosh S., Bao W., Calizo I., Teweldebrhan D., Miao F., Lau C.N. (2008). Superior thermal conductivity of single-layer graphene. Nano Lett..

[58] Zhou H., Zhu J., Liu Z., Yan Z., Fan X., Lin J., Wang G., Yan Q., Yu T., Ajayan P.M. (2014). High thermal conductivity of suspended few-layer hexagonal boron nitride sheets. Nano Res..

[59] Zhang Y.Y., Pei Q.X., Liu H.Y., Wei N. (2017). Thermal conductivity of a h-BCN monolayer. Phys. Chem. Chem. Phys..

[60] Schelling P.K., Phillpot S.R., Keblinski P. (2002). Comparison of atomic-level simulation methods for computing thermal conductivity. Phys. Rev. B.

[61] Smith B., Vermeersch B., Carrete J., Ou E., Kim J., Mingo N., Akinwande D., Shi L. (2017). Temperature and thickness dependences of the anisotropic in-plane thermal conductivity of black phosphorus. Adv. Mater..

[62] Hong Y., Zhang J., Huang X., Zeng X.C. (2015). Thermal conductivity of a two-dimensional phosphorene sheet: A comparative study with graphene. Nanoscale.

[63] Chen J., Zhang G., Li B. (2013). Substrate coupling suppresses size dependence of thermal conductivity in supported graphene. Nanoscale.

[64] Li X., Zhang J., Puretzky A.A., Yoshimura A., Sang X., Cui Q., Li Y., Liang L., Ghosh A.W., Zhao H. (2019). Isotope-engineering the thermal conductivity of two-dimensional MoS_2_. Acs Nano.

[65] Netto A., Frenklach M. (2005). Kinetic Monte Carlo simulations of CVD diamond growth—Interlay among growth, etching, and migration. Diam. Relat. Mater..

[66] Mortazavi B., Podryabinkin E.V., Novikov I.S., Rabczuk T., Zhuang X., Shapeev A.V. (2021). Accelerating first-principles estimation of thermal conductivity by machine-learning interatomic potentials: A MTP/ShengBTE solution. Comput. Phys. Commun..

[67] Aiyiti A., Bai X., Wu J., Xu X., Li B. (2018). Measuring the thermal conductivity and interfacial thermal resistance of suspended MoS 2 using electron beam self-heating technique. Sci. Bull..

